# Survey on retinopathy of prematurity (ROP) in Italy

**DOI:** 10.1186/1824-7288-39-43

**Published:** 2013-07-09

**Authors:** Cesarina Borroni, Carla Carlevaro, Sabrina Morzenti, Elena De Ponti, Valentina Bozzetti, Vito Console, Salvatore Capobianco, Paolo E Tagliabue

**Affiliations:** 1 Neonatology and Neonatal Intensive Care Unit Fondazione MBBM, Monza, Italy; 2 Ophtalmology Department, S. Gerardo Hospital, Monza, Italy; 3 Medical Physics Department, S. Gerardo Hospital, Monza, Italy; 4 Executive board of the Italian “ROP Study Group”, Torino, Italy; 5 Pediatric Department, Santobono-Annunziata-Pausilipon-Hospital, Naples, Italy

**Keywords:** Retinopathy of prematurity (ROP), Posterior ROP, Plus-disease, Laserterapy, Extremely preterm infants

## Abstract

**Background:**

This study aims to investigate the incidence and the relative risk factors of retinopathy of prematurity (ROP) and posterior-ROP (P-ROP): ROP in Zone I and posterior Zone II, as well as to analyze the occurrence of surgical treatment of ROP and to evaluate the short term outcome of the disease in Italy.

**Methods:**

It is a prospective multicenter observational study; all infants with a birth weight (BW) ≤ 750 g and/or a gestational age (GA) ≤27 weeks born between January 1^st^ 2008 and December 31^st^ 2009 in 25 III level Italian neonatal intensive care units were eligible for the study.

**Results:**

421 infants were examined: 265 (62.9%) developed ROP and 102 (24.2%) P-ROP.

Following the multivariate analysis erythropoietin-therapy (p < 0.0001) and intraventricular hemorrhage (IVH) (p = 0.003) were significantly associated with ROP while gestational age ≤24 weeks (p = 0.011) and sepsis (p = 0.002) were associated with the onset of P-ROP. Eighty nine infants (34%) required surgical treatment; following the multivariate analysis P-ROP was an independent factor associated with the need of surgical treatment (p < 0.0001). A favorable outcome was reported in 251 (94.7%) newborns affected by ROP. Adverse outcome occurred in 14 patients: all of them underwent surgery and showed P-ROP.

**Conclusions:**

P-ROP is the most aggressive type of ROP. It associates with lower GA and sepsis. Obstetricians and Neonatologists must focus on the reduction of severe preterm births and on the prevention of neonatal early and late onset sepsis in order to reduce the incidence of P-ROP.

## Background

Retinopathy of prematurity (ROP) is a vasoproliferative disorder of the retina that may result in a significant loss of vision and even blindness. In recent decades the survival rate of extremely preterm infants has improved dramatically [1]. Since extremely preterm infants are at high risk of developing ROP, the number of infants with severe ROP has risen considerably in the last few years. Currently, ROP is one of the most common causes of childhood blindness [2].

In 1951, Campbell first highlighted the involvement of supplemental oxygen in the pathogenesis of ROP; the role of oxygen was subsequently reconfirmed by other authors [3,4]. During the first stage of the pathogenesis of ROP, hyperoxia suppresses the activity of the vascular endothelial growth factor (VEGF) and alters the normal vascularization of the retina due to vaso-constriction and vaso-obliteration of the existing immature vessels. In the second phase of ROP, upregulation of VEGF and other growth factors, triggered by hypoxia, induces vascular overproliferation. Thus both hypoxia and hyperoxia are involved in the pathogenesis of ROP [5].

Fifty percent of extremely preterm infants show clinical signs of ROP, although this percentage varies widely. Complete recovery rate is approximately 85% [6].

Several papers have described the risk factors for ROP and have provided recommendations for the prevention of the disease [7,8]. The American Academy of Pediatrics, the American Academy of Ophthalmology and the American Association of Pediatric Ophthalmology and Strabismus have established the screening instructions that are currently used in neonatal intensive care units (NICUs) [9-12]. According to these studies all preterm infants born at less than 30 weeks of gestation and/or with a birth-weight less than 1500 g should undergo an indirect ophthalmoscopy of dilated eyes. The ophthalmoscopy should start from the 31^st^ week of postconceptional age or from the 28^th^ day of life. The CRYO-ROP report has defined the criteria of the “threshold ROP”; the ETROP data, published in December 2003, demonstrated a benefit of earlier treatment compared with conventional management; babies who meet ETROP criteria should be considered eligible for surgical treatment (cryo-therapy, argon or diode laser-therapy) [12,13].

The aim of our prospective multicenter cohort study is to investigate the incidence and the relative risk factors of ROP and P-ROP, the incidence of surgical treatment and to evaluate the short-term outcome of the disease in Italy.

## Methods

This study, promoted by the “Italian ROP study group”, involved a cohort of infants born between January 1^st^ 2008 and December 31^st^ 2009 with a birth weight (BW) ≤ 750 g and/or a gestational age (GA) ≤27 weeks, in 25 III level Italian NICUs. Gestational age was confirmed by fetal ultrasound. In order to reduce differences in the neonatal assistance, the newborns admitted to the III level NICUs 6 hours following birth were excluded; newborns who died before discharge and newborns affected by major congenital malformations were also excluded.

The following data for each infants were recorded on a specific database: gender, race, premature rupture of membranes, chorioamnionitis, use of prenatal steroids, place of birth (inborn or outborn), fifth minute Apgar score, days of mechanical ventilation, days of parenteral nutrition, doses of erythropoietin (EPO) and blood transfusions.

The following complications of preterm birth were registered:

– intraventricular hemorrhage (IVH): any grade according to the Volpe classification [14]

– periventricular leukomalacia (PVL): evaluated at any age by ultrasounds (cystic periventricular white matter lesions) or by Magnetic Resonance Imaging (periventricular high-intensity areas on T2-weighted images and atrophy of the cerebral white matter predominantly at the peritrigonal region)

– respiratory distress syndrome (RDS): oxygen requirement increasing during the first 24 hours, typical radiological pattern such as reduced air content, reticulogranular pattern of the lung air and bronchogram

– patent ductus arteriosus (PDA): clinical evidence of left to right PDA shunt or ecocolorDoppler evidence of PDA with evidence of left to right ductal shunting

– early or late onset sepsis:

• early: bacterial and/or fungal infections, diagnosed by means of a positive culture of blood and/or cerebrospinal fluid, within the first three days of life,

• late sepsis: if diagnosed, after the third day of life

– moderate or severe BPD, in accordance with the definition of Jobe and Bancalari [15]

• moderate BPD is determined as the need for < 30% oxygen at 36 week postmenstrual age (PMA) or discharge whichever comes first

• severe BPD is determined as the need for ≥ 30% oxygen and/or positive pressure (NCPAP or IPPV) at 36 week PMA or discharge whichever comes first

This is a prospective non-interventional survey, the participating neonatal intensive care unit assisted the newborns in accordance with their own protocols; therefore the management and the care of the patients in each center varied one from the other.

Only the eye examinations were standardized: all the participating centers have a staff of pediatric ophthalmologists. Following pupil dilation with cyclopentolate hydrochloride 0.2% and phenylephrine hydrochloride 1%, pediatric ophthalmologists performed indirect ophthalmoscopies using scleral depression, if required, to visualize the retinal periphery***,*** at weekly intervals, starting from the 4^th^ week of life until complete retinal vascularization occurred [12].

Ophthalmologists diagnosed ROP according to the ICROP report and classified the severity of ROP on the basis of:

Zone:

– Posterior: ROP in Zone I and posterior Zone II (P-ROP) [16]

– other zones: ROP in other Zones (OZ-ROP)

Presence of pre-plus (p-PD) or plus disease (PD) [12].

The classification based on Zone and on the presence of plus or pre-plus disease was chosen in order to reduce the variability in the classification of ROP among the various centers participating in the trial.

In case of surgical intervention, ophthalmologists reported the type of intervention carried out (Laser-therapy, Cryo-therapy) and the number of ablative surgical treatments performed. The outcome was defined “adverse” if total or partial retinal detachment or macular fold occurred, while normal anatomy (no detachment or macular fold) was defined as “a favorable outcome”.

Univariate and multivariate logistic regression models were used to estimate the odds ratios (ORs) and the p-values for the association between ROP and the clinical parameters of the patients. Initially, the models were used to evaluate the independent predicting factors for developing ROP of any stage; the independent parameters associated with the evolution of the disease were also studied. Moreover, ORs were used to describe the characteristics of the infants that were candidates for the laser treatment. Sample size was calculated to detect a difference equal or greater than 15% in relative frequencies of different groups, with 5% significance level and a power of 80%.

A P value <0.05 was considered significant. All statistical analysis was performed with Stata software 9.0 (Stata Corporation, 1999, Texas, U.S.A.).

## Results

The study involved 421 VPI; the spreadsheets were not completed in full for each newborn, with a rate of incompleteness for each parameter between 4% and 15%. Birth weight, gestational age and ROP description were considered mandatory, moreover newborns with more than 10% missing data were not included in the sample.

Table 1 shows the distribution of birth weight and gestational age, and the clinical features of the study population.

**Table 1:** Population characteristics

**Parameters**	**N° of newborns**	**%**	**Parameters**	**N° of newborns**	**%**
GA (weeks)			RDS	384	99.7%
22–23 - 24	92	22.6%	PDA	251	65.0%
25	102	24.2%	IVH	167	43.5%
26	134	31.8%	LPV	99	26.7%
≥ 27	90	21.4%	Fungal Sepsis	46	11.4%
			Bacterial Sepsis	178	44.2%
BW (g)			BPD	208	54.9%
300–450	9	2.1%	Blood transfusions	357	89.9%
451–600	70	16.6%	EPO therapy	210	55.6%
601–750	167	39.7%	Mechanical ventilation ( > 7 days)	258	69.2%
> 750	175	41.6%	NPT ( > 7 days)	303	80.6%
Male	182	45.5%	ROP	265	62.9%
Inborn	358	88.0%	P - ROP	102	24.2%
APGAR score 5th minute ≤ 7	176	47.7%	Preplus disease	55	13.1%
PROM	122	31.9%	Plus disease	79	18.8%
Chorioamnionitis	93	27.8%	Laser treatment	89	21.1%
Antenatal steroids	331	84.0%	Adverse outcome	14	3.3%

All infants underwent the eye examinations in accordance with the guidelines. Ophthalmological screening was started at an average of 30 weeks of postmenstrual age. ROP was diagnosed in 265 newborns (62.9%). At the univariate analysis (Table 2) GA (p = 0.008), BW (p = 0.007), PDA (p =0.002), IVH (p = 0.001), early and late onset sepsis (p = 0.001), BPD (p = 0.002) and EPO therapy (p < 0.001), showed a significant association with the occurrence of ROP.

**Table 2:** Association between ROP and neonatal parameters: logistic univariate analysis

**Parameters**	**ROP**	**No ROP**	**Univariate analysis**	
	**n newborns (%)**		**OR**	**p-value**
GA ≤ 24 weeks	71 (75%)	24 (25%)	2.01	0.008
BW ≤ 750 g	168 (68%)	78 (32%)	1.73	0.007
Male gender	111 (45%)	71 (46%)	0.96	0.848
Inborn	217 (86%)	141 (90%)	0.68	0.238
APGAR score 5th minute ≤ 7	125 (56%)	72 (49%)	1.31	0.205
PROM	78 (34%)	44 (29%)	1.22	0.380
Chorioamnionitis	60 (31%)	33 (23%)	1.47	0.130
Antenatal steroids	198 (81%)	133 (88%)	0.59	0.085
RDS	234 (100%)	150 (100%)	Not applicable	
PDA	167 (66%)	84 (34%)	1.96	0.002
IVH	118 (71%)	49 (29%)	2.10	0.001
LPV	44 (19%)	55 (38%)	1.22	0.543
Sepsis	134 (70%)	57 (30%)	2.02	0.001
BPD	142 (61%)	66 (45%)	1.94	0.002
Blood transfusions	217 (90%)	140 (90%)	0.93	0.833
EPO therapy	147 (70%)	63 (30%)	2.39	< 0.0001
Mechanical ventilation (>7 days)	157 (71%)	101 (67%)	1.20	0.432
NPT ( > 7 days)	185 (83%)	118 (78%)	1.37	0.234

**Figure 1:**
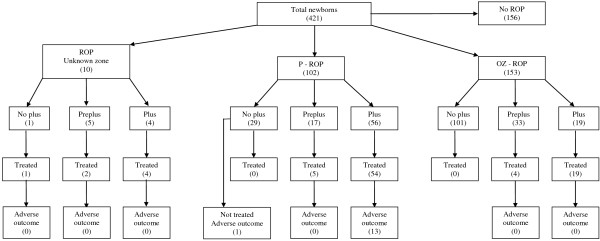
Outcomes of the enrolled newborns.

Following the multivariate analysis only EPO-therapy (p < 0.0001) and IVH (p = 0.003) were significantly associated with ROP (Table 3). P-ROP was diagnosed in 102 cases (24.2%); the ROP Zone was not reported in 10 cases; 7 of these infants underwent surgical treatment and all of them had a favorable outcome (Figure 1).

**Table 3:** Association between ROP and neonatal parameters: logistic multivariate analysis

**Parameters**	**ROP**	**No ROP**	**Multivariate analysis**	
	**n newborns (%)**		**OR**	**p-value**
GA ≤ 24 weeks	71 (75%)	24 (25%)	1.58	0.147
BW ≤ 750 g	168 (68%)	78 (32%)	1.63	0.053
PDA	167 (66%)	84 (34%)	1.21	0.455
IVH	118 (71%)	49 (29%)	2.10	0.003
Sepsis	134 (70%)	57 (30%)	1.43	0.135
BPD	142 (68%)	66 (32%)	1.32	0.249
EPO	147 (70%)	63 (30%)	2.93	< 0.0001

Gestational age (p = 0.002), sepsis (p = 0.002) and BPD (p = 0.02) were significantly associated with P-ROP. At the multivariate analysis, gestational age ≤ 24 weeks (p = 0.011) and sepsis (p =0.002) appeared to be independent factors leading to P-ROP (Table 4).

**Table 4:** Association between P-ROP or treated newborn and neonatal parameters: logistic univariate and multivariate analysis

**Parameters**	**P - ROP**	**OZ - ROP**	**Univariate analysis**		**Multivariate analysis**	
	**n newborns (%)**		**OR**	**p-value**	**OR**	**p-value**
GA ≤ 24 weeks	37/71 (52.1%)	29/71 (40.8%)	2.43	0.002	2.30	0.011
Sepsis	60/134 (44.8%)	68/134 (50.7%)	2.35	0.002	2.64	0.002
BPD	57/142 (40.1%)	79/142 (55.6%)	2.01	0.020	1.53	0.182
**Association between zones and significant neonatal parameters: univariate and multivariate logistic analysis**						
**Parameters**	**Treatment**	**No treatment**	**Univariate analysis**		**Multivariate analysis**	
	**n newborns (%)**		**OR**	**p-value**	**OR**	**p-value**
P - ROP	59/89 (66.3%)	35/142 (24.6%)	7.84	< 0.0001	7.30	< 0.0001
EG ≤24 weeks	33/89 (37.1%)	33/142 (23.2%)	1.95	0.024	0.97	0.946
BW ≤ 750 g	69/89 (77.5%)	79/142 (55.6%)	2.75	0.001	2.07	0.054
Sepsis	54/89 (60.7%)	61/142 (42.9%)	2.36	0.003	1.58	0.188
**Association between laser treatment and significant neonatal parameters: univariate and multivariate logistic analysis**						

PD was diagnosed in 79 newborns; 56 of them were affected by P-ROP (55%) and 19 were affected by OZ-ROP, (in 4 infants ROP Zone was not reported) (Figure 1).

p-PD was diagnosed in 17 newborns with P-ROP, in 33 infants with OZ-ROP and in 5 patients the zone of ROP was unknown (Figure 1). These differences were statistically significant (p < 0.0001).

Eighty-nine infants underwent surgery (34% of the 265 newborns with ROP); 59 of them (66.3%) showed a P-ROP and 23 (25.8%) an OZ-ROP (Figure 1). This difference was statistically significant (p <0.0001).

Zone of ROP, gestational age, birth weight and sepsis were significantly associated with need of surgery at the univariate analysis; following the multivariate analysis only P-ROP was an independent factor associated with the need for surgical treatment (p < 0.0001). Birth weight below 750 g showed a borderline significance (p = 0.054), while gestational age (p = 0.946) and sepsis (p = 0.188) were not statistically related to the requirement of laser therapy. The use of EPO was not associated with surgical treatment: 54% of the surgically treated infants and 68.2% of the non-treated infants received EPO.

PD was diagnosed in 87.5% of the surgically treated infants and p-PD in 12.5% of cases. None of the newborns without PD or p-PD underwent surgery. One hundred seventy newborns with ROP were not surgically treated: PD was diagnosed in only 2 and p-PD in 41 (21.1%)(Figure 1).

Ophthalmologists performed surgical therapy between the 32^nd^ and 34^th^ weeks of gestation: laser-therapy, argon laser 532 nm or diode laser 810 nm, and cryotherapy. In all cases the laser-therapy was performed; in 8 cases cryotherapy was attempted as additional treatment. Four newborns with P-ROP required vitrectomy following laser and cryotherapy (1.5% of all newborn with ROP). All patients showed P-ROP in Zone 1. The outcome was evaluated as favorable in 251 non-treated infants (94%) and in 68 (86.7%) of surgically treated infants. Adverse outcomes occurred in 14 patients (3.3% of total population, 5.3% of infants with ROP and 14.6% of surgically treated infants): 11 had bilateral and 3 unilateral retinal detachment. All 14 had P-ROP and underwent surgery (Figure 1).

## Discussion

ROP widely affected extremely premature infants: the occurrence of P-ROP represented the main risk factor for surgical treatment. A considerable number of newborns required surgery; laser therapy was mostly effective and adverse outcomes were rare.

Our incidence data differ from those of the Cryotherapy for Retinopathy of Prematurity Cooperative Group trial and from those of the ETROP study [9,17]; these studies reported a higher incidence of ROP but they were conducted prior to the introduction of the current guidelines to prevent ROP [18].

Other studies showed results similar to ours [19-21]. The 2009 Vermont-Oxford Network Report showed an incidence of ROP superimposable on our results [22].

In the multivariate analysis EPO therapy and IVH significantly associated with the incidence of ROP; birth weight showed only a borderline association. EPO therapy was a risk factor for ROP, but not for P-ROP. Shah et al. did not find any significant difference between EPO therapy and the presence or severity of ROP [23]. Before the “EPO era”, blood transfusions were more common in NICUs and some researchers considered them as a risk factor for developing ROP [24,25]. Suk et al. in a multiple regression model found that blood transfusions were a significant independent risk factor for the development of ROP. Currently it is not clear if the therapy with EPO is advantageous compared to the blood transfusions in treating anemic preterm infants [26]. Schneider et al. showed that EPO therapy does not increase the number and the severity of ROP cases, while it may reduce the number of blood transfusions [27]. Recently, in a mouse model of proliferative retinopathy, Chen et al. studied the role of EPO in suppression of retinal neovascularization; they proposed the hypothetic intravitreal use of EPO in proliferative retinopathy [28].

In our study EPO administration was a factor involved in the development of ROP, while it was not a factor related to the severity of the disease: it was not significantly associated to P-ROP and to the necessity of surgical intervention. GA ≤ 24 weeks and sepsis were significant risk factors for developing P- ROP. Even though gestational age was not directly related to the ROP, it triggered P-ROP and therefore was one of the main causes of the more severe type of the disease [29].

It is noteworthy that BPD was not associated with the ROP, nor with the more aggressive kind of ROP, even though the use of oxygen is particularly involved in ROP pathogenesis. This may arise from the use of more accurate targets of oxygen saturation during oxygen-therapy, both in the first weeks of life, when hyperoxia is dangerous, and also during the following weeks, when hypoxia induces neovascularization. Some authors recommend monitoring oxygen therapy on the basis of gestational age and postnatal age: low-oxygen levels during early phases of life and high levels in the later phases [30].

Our data on the risk factors for P-ROP are consistent with the pathogenetic model recently proposed by Lee and Damman: in their review they concluded that exposure to perinatal infection/ inflammation is associated with an increased risk for ROP. Circulating products of infection and/or inflammation might directly damage the developing retina; moreover inflammation and/or oxidative stress might increase the risk of oxygen-associated ROP. This means that prenatal, perinatal, and postnatal systemic inflammation could sensitize the pre-ROP retina for subsequent insults, setting the stage for what is now called phase I and phase II of ROP pathogenesis. The authors concluded that strategies for targeting inflammatory responses might help in reducing the risk for ROP in extremely preterm infants [31].

In our study laser therapy was performed in all infants requiring surgery; most of these infants had a P-ROP. PD represented the main indicator for surgical intervention as also reported in the ICROP study [11].

In almost all cases, the infants with ROP associated with PD underwent surgical intervention; only 2 out of 75 newborns with PD were not treated with laser therapy. Moreover, occurrence of p-PD represented an indication for surgical intervention; although it was more evident in the newborns affected by P-ROP than in OZ-ROP there was probably an overtreatment in some cases. PD was significantly higher in P-ROP than in OZ-ROP; therefore infants with P-ROP underwent surgery more frequently than infants with OZ-ROP. These observations confirm the severity of P-ROP.

The percentage of children treated with laser therapy in our population was equal to the percentage reported in a Malaysian study and double the number reported in a Swedish paper [20,21]. This variability of data may be due to the differences of the populations involved. As Darlow et al. in the Australian and New Zealand Neonatal Network argued, the discrepancy between the data reported in scientific literature may be attributed to a wide variability in the classification of ROP [32]. For this reason we classified the severity of ROP on the basis of zone and presence of PD or p-PD. Our protocol allowed us to classify ROP disease more clearly without the inter-individual differences of ophthalmologists.

Treatment was performed between the 32^nd^ and the 34^th^ weeks of gestation. The majority of surgically treated infants had a favorable outcome; this is encouraging even though Ruth Axer-Siegel and coll. reported 92.3% favorable outcomes following laser therapy in a cohort of 100 neonates, which is slightly better than our data [33]. It is important to underline that all 14 infants with an adverse outcome were ≤24 weeks gestation, each one underwent almost one episode of sepsis and was affected by P- ROP: this confirms once more that P-ROP represents the most severe form of ROP and it is associated with the worst short term outcome.

## Conclusion

In order to prevent severe ROP in very preterm infants, a multidisciplinary strategy is necessary: obstetricians should prevent preterm births and intrauterine infections; neonatologists should reduce the occurrence of septic diseases and should carefully monitor infant oxygen exposure. In all NICUs, a pediatric ophthalmologist should periodically evaluate all very preterm infants from the 28^th^ day of postnatal age. The follow-up of infants with Zone I and Zone II posterior ROP needs to be particularly scrupulous since these infants are at high risk of surgical intervention and adverse outcome.

## Abbreviations

OZ-ROP: ROP in any other Zones; PD: Presence of plus disease; p-PD: Presence of pre-plus disease; P-ROP: ROP in posterior Zone (Zone I and posterior Zone II).

## Competing interests

The authors declare that they have no competing interests.

## Authors’ contributions

CB: Conception and design of the study, study coordination, analysis and interpretation of data, manuscript draft. CC: Analysis and interpretation of data, manuscript draft (ophthalmological section). EDP– SM: Statistical analysis. VB: Interpretation of data, manuscript draft. VC: Conception and design of the study. SC: Conception and design of the study. PET: interpretation of data, manuscript draft, critical revision. All authors read and approved the final manuscript.
